# Supplementary material for: p53 Transactivation Domain Mediates Binding and Phase Separation with Poly-PR/GR
Source: Int J Mol Sci. 2021 Oct 22;22(21):11431. doi: 10.3390/ijms222111431 (PMC8583712; doi:10.3390/ijms222111431)
Supplement: Supplementary file 1 [file ijms-22-11431-s001.zip › ijms-1401019 supplementary.pdf]

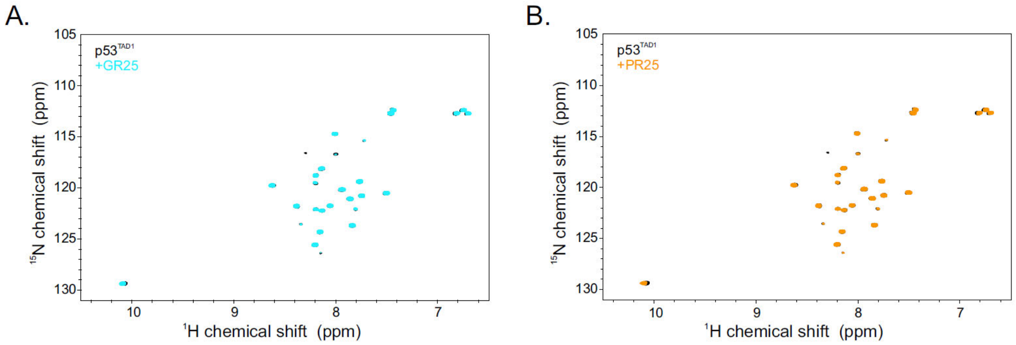

Supplementary Figure S1. p53^{TAD1} does not bind to PR25/GR25 on its own. (**A, B**) ^{1}H-^{15}N HSQC spectrum of ^{15}N-labeled p53^{TAD1} at 50 µM in the absence (black) and presence of one stoichiometric equivalent of GR25 (cyan in **A**) and of PR25 (orange in **B**).

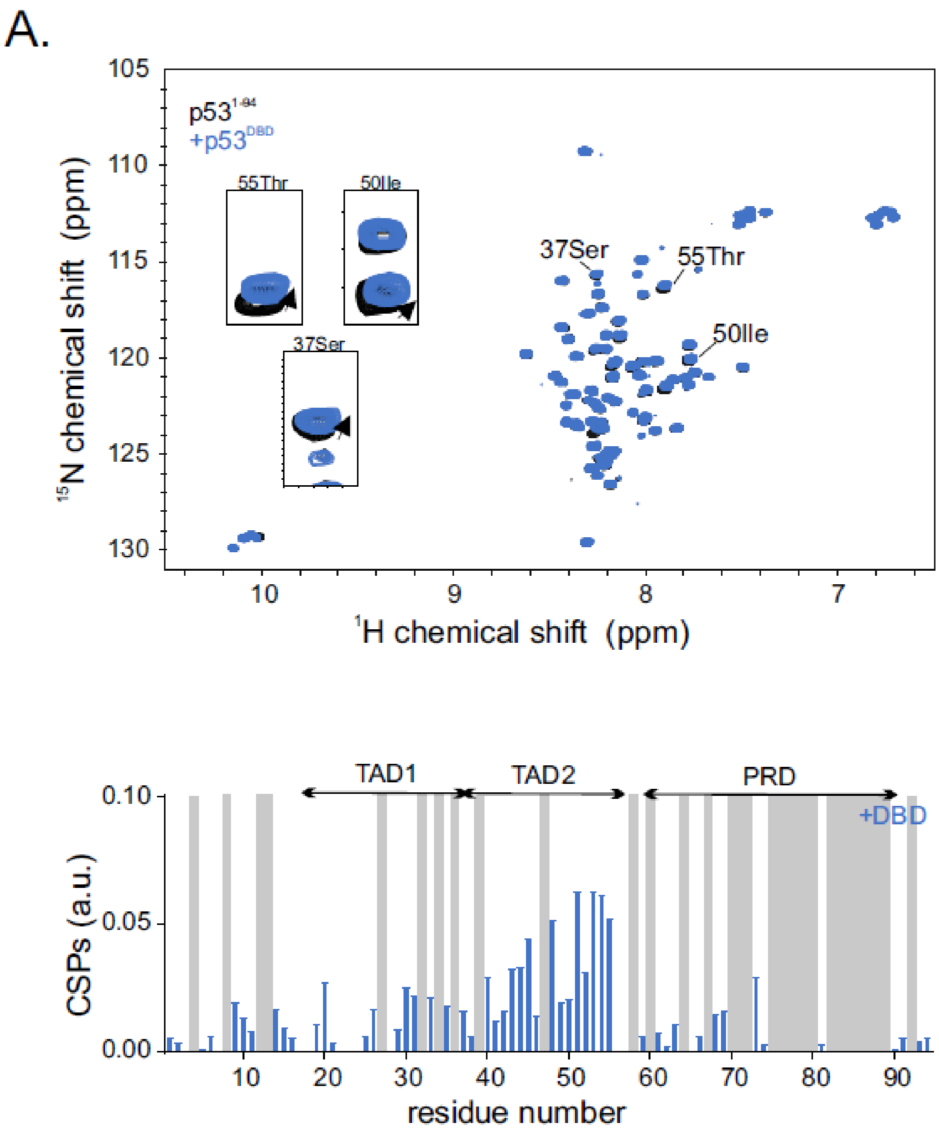

Supplementary Figure S2. **p53^{TAD2} binds to p53^{DBD}.** (**A**) ^{1}H-^{15}N HSQC spectrum of ^{15}N-labeled p53$^{1\text{-}94}$ at 50 µM absence (black) and presence of one stoichiometric equivalent of p53^{DBD} (blue; upper panel). Corresponding CSPs are shown in a bar-plot at the bottom panel. Unassigned residues are indicated in grey.
